# Determination of Cytotoxic Activity of *Sanguinaria canadensis* Extracts against Human Melanoma Cells and Comparison of Their Cytotoxicity with Cytotoxicity of Some Anticancer Drugs

**DOI:** 10.3390/molecules26061738

**Published:** 2021-03-20

**Authors:** Tomasz Tuzimski, Anna Petruczynik, Tomasz Plech, Barbara Kaproń, Anna Makuch-Kocka, Małgorzata Szultka-Młyńska, Justyna Misiurek, Bogusław Buszewski

**Affiliations:** 1 Department of Physical Chemistry, Medical University of Lublin, Chodźki 4a, 20-093 Lublin, Poland; 2 Department of Inorganic Chemistry, Medical University of Lublin, Chodźki 4a, 20-093 Lublin, Poland; justyna.misiurek@umlub.pl; 3 Department of Pharmacology, Medical University of Lublin, Chodźki 4a, 20-093 Lublin, Poland; tomasz.plech@umlub.pl (T.P.); anna.makuch-kocka@umlub.pl (A.M.-K.); 4 Department of Clinical Genetics, Medical University of Lublin, Radziwiłłowska 11, 20-080 Lublin, Poland; barbara.kapron@umlub.pl; 5 Department of Environmental Chemistry and Bioanalytics, Faculty of Chemistry, Nicolaus Copernicus University, Gagarina 7, 87-100 Torun, Poland; szultka.malgorzata@wp.pl (M.S.-M.); bbusz@chem.umk.pl (B.B.)

**Keywords:** *Sanguinaria canadensis*, various vegetation steps, isoquinoline alkaloids, cytotoxic activity, human melanoma cells

## Abstract

Melanoma is an enormous global health burden, and should be effectively addressed with better therapeutic strategies. Therefore, new therapeutic agents are needed for the management of this disease. The aim of this study was the investigation of cytotoxic activity of some isoquinoline alkaloid standards and extracts obtained from *Sanguinaria canadensis*—collected before, during, and after flowering—against three different human melanoma cells (A375, G361, SK-MEL-3). The cytotoxicity of these extracts was not previously tested on these melanoma cell lines. Determination of alkaloid contents was performed by HPLC-DAD using Polar RP column and mobile phase containing acetonitrile, water, and 1-butyl-3-methylimidazolium tetrafluoroborate. The cytotoxicity of alkaloid standards was investigated by determination of cell viability and calculation of IC_50_ values. Significant differences were observed in the alkaloids content and cytotoxic activity of the extracts, depending on the season of collection of the plant material. In the *Sanguinaria canadensis* extracts high contents of sanguinarine (from 4.8543 to 9.5899 mg/g of dry plant material) and chelerythrine (from 42.7224 to 6.8722 mg/g of dry plant material) were found. For both of these alkaloids, very high cytotoxic activity against the tested cell lines were observed. The IC_50_ values were in the range of 0.11–0.54 µg/mL for sanguinarine and 0.14 to 0.46 µg/mL for chelerythrine. IC_50_ values obtained for *Sanguinaria canadensis* extracts against all tested cell lines were also very low (from 0.88 to 10.96 µg/mL). Cytotoxic activity of alkaloid standards and *Sanguinaria canadensis* extracts were compared with the cytotoxicity of anticancer drugs—etoposide, cisplatin, and hydroxyurea. In all cases except the one obtained for cisplatin against A375, which was similar to that obtained *for Sanguinaria canadensis* after flowering against the same cell line, IC_50_ values obtained for anticancer drugs were higher than the IC_50_ values obtained for sanguinarine, chelerythrine, and *Sanguinaria canadensis* extracts. Our results showed that *Sanguinaria canadensis* extracts and isoquinoline alkaloids, especially sanguinarine and chelerythrine, could be recommended for further in vivo experiments in order to confirm the possibility of their application in the treatment of human melanomas.

## 1. Introduction

Currently cancers are an enormous global health problem and the second leading cause of mortality worldwide [1]. One of the major factor responsible for the failure of chemotherapy is the development of multi-drug resistance. Skin cancer evolved to be one of the most common malignant cancer, with an increase of new cases annually. Standard treatment can prove effective for patients with early stage of the diseases. However, patients with the advanced disease typically respond poorly and progress rapidly. New, more effective therapies are therefore an urgent unmet medical need for these patients. Extensive research in past few decades led to the development of many synthetic antineoplastic agents. However, they often have serious side effects and are expensive. Natural compounds are often less toxic, show fewer side effects, are easily available and are cost effective [1]. These compounds have multi-targeted approach, specifically or non-specifically, on various signal transduction pathways. Natural compounds can also be used in adjunctive chemotherapy with conventional anticancer therapy, such as surgical removal of tumor and radiation therapy, which might pose enhanced promising effects than single therapy. Natural products and their synthetic analogues are a major source of therapeutic agents, and were used for centuries to treat a variety of human ailments [2].

Melanoma is the most belligerent form of skin cancer with an eminent metastatic potential. It is an aggressive, therapy-resistant malignancy of melanocytes [3]. The incidence of melanoma is steadily increasing, resulting in an increasing public health problem. Treatment that improves overall survival in patients with advanced metastatic melanoma is not yet developed, therefore, a new treatment option is urgently needed. Therefore, search of new compounds that can stop the proliferation of melanoma cells is an important target in the field of cancer research. Plant secondary metabolites, including isoquinoline alkaloids, also belong to the group of molecules with antiproliferative potential [4].

Isoguinoline alkaloids exhibit many types of biological activities such as anti-hypertensive, anti-inflammatory, anti-diabetics, antioxidant, antidepressant, and hepatoprotective. Significant anticancer activity of these compounds was also reported [4,5].

*Sanguinaria canadensis* contain large amounts of sanguinarine, which proved to be a candidate with developmental promise in the treatment of various cancers [6]. Lee et al. described cytotoxic activity of sanguinarine against human oral squamous cell carcinoma KB cells [7]. They investigated the mechanism of sanguinarine anticancer activity and concluded that sanguinarine treatment increased DR5/TRAILR2 (death receptor 5/TRAIL receptor 2) expression and enhanced the activation of caspase-8 and cleavage of its substrate, Bid. The alkaloid also induced the mitochondrial translocation of pro-apoptotic Bax, mitochondrial dysfunction, cytochrome c release to the cytosol, and activation of caspase-9 and -3. Sanguinarine also suppressed the phosphorylation of phosphoinositide 3-kinase (PI3K). Sanguinarine exhibited cytotoxic activity against Jurkat and Molt-4human leukemic cells in vitro [8]. They observed that sanguinarine induces caspase-dependentapoptosis in human leukemic cells, which is regulated by H_2_O_2_-dependent ceramide upregulation, which in turn activates protein phosphatase 1, leading to Akt dephosphorylation. Rahman et al. investigated in vitro anticancer activity of sanguinarine against various prostate cancer cell lines [9]. The investigations confirmed that sanguinarine induces apoptosis in prostate cancer cells. They stated that sanguinarine-induced apoptosis is regulated by H_2_O_2_-mediated ceramide generation, extracellular signal-regulated kinase1/2 phosphorylation, and prostate apoptosis response-4 cleavage. Sanquinarine was also tested as a cytotoxic agent against human hepatocellular carcinoma HeLa cells [2]. The authors found that sanguinarine promotes oxidative stress-mediated apoptotic cell death. They described that sanguinarine inhibited thioredoxin reductase, which leads to accumulation of the oxidized thioredoxin, elicits oxidative stress, and finally promotes apoptosis of cancer cells. Sanguinarine inhibits cell viability and induces cell death by apoptosis in both human breast cancer (MDA-MB-231) and mouse (A17) cells [10]. Performed experiments demonstrated that oral administration of sanguinarine reduced the development and growth of A17 transplantable tumors in FVB syngeneic mice. Authors identified sanguinarine as a potent inhibitor of dihydrofolate reductase, which is able to impair enzyme activity even in methotrexate-resistant MDA-MB-231 cells. Lymphoblastic leukemia RS4;11, SupB15, REH, and 697 cells lines treated with sanguinarine exhibited significant inhibition of cell viability through induction of apoptotic cell death [11]. Sanguinarine also inhibited the viability of human promyelocytic leukemia HL60 cell line [12]. The data obtained in this research indicated that sanguinarine induced apoptosis through a caspase-3/7-dependent mechanism involving the phosphorylation of p38—a protein phosphatase 2C α substrate that partially inhibited SB203580—that was involved in the phospho-p38 protein in investigated cancer cells. Dong et al. demonstrated in vitro antiproliferative activity of sanguinarine against gastric cancer BGC-823 cell line [13]. The authors concluded that sanguinarine induced proliferation inhibition in the investigated gastric cancer cells by inhibiting miR-96-5p and miR-29c-3p expression and activating the MAPK/JNK signaling pathway. Sanguinarine also exhibited the in vitro cytotoxic activity against the tumor cells of three lines of human prostate carcinomas, LNCaP clone FGC (ATCC^®^ CRL-1740™), DU 145 (ATCC^®^ HTB-81™), and PC3 (ATCC^®^ CRL-1435™) [14]. In the experiments, sanguinarine was introduced into the pH-sensitive liposome to increase the effectiveness of its therapeutic effects. Inhibition of human LoVo, SW480, and Caco-2 colorectal carcinoma cells viability, migration, and metastasis by sanguinarine was also confirmed [15]. The obtained results indicated the suppression of the Wnt/ β-catenin signaling by sanguinarine, which further upregulated the level of E-cadherin and decreased the expression of N-cadherin, Snail, metalloproteinase 2, and metalloproteinase 9, and finally prevented epithelial to mesenchymal transition. Sanguinarine also inhibited the viability of non-small cell lung carcinomas (A549, H460, H1299, H1650, and SPC-A1) [16] and pancreatic cancer cell lines (AsPC-1 and PANC-1) [17].

In vitro anticancer activity of sanguinarine and chelerythrine were investigated on breast cancer MCF-7, breast cancer MDA-MB231, colon cancer HCT116, colon cancer SW480, and ovarian cancer OVCAR-3 cell lines [18]. The authors proposed an anticancer activity model in which the generation of lethal amounts of hydrogen peroxide is explained by an enzyme-catalyzed redox cycling between the reduced and oxidized forms of the phenanthridines.

Antiproliferative activity of sanguinarine and cheleritrine was also investigated in the therapy of melanoma cancer [3]. The obtained results demonstrated that the antitumoral effect of sanguinarine relies on its activity on several critical steps in primary tumor progression, including cell proliferation and angiogenesis. In vitro cytotoxic activity of sanguinarine and cheleritrine was obtained against B16 melanoma 4A5 cells, a cell line derived from subcutaneously inoculated B16F0 tumors in the C57BL/6 strain mouse, and human melanoma A375 cells. Antiproliferative effects of isoquinoline alkaloids—sanguinarine, chelerythrine, chelidonine, sanguilutine, and chelilutine was investigated on malignant melanoma A-375 and SK-MEL-2 cell lines. Cytotoxicity of sanguinarine were also investigated on mouse melanoma K1735-M cell line [19]. The data obtained in the investigations suggest that sanguinarine acts as a DNA damaging agent, also showing collateral damage to mitochondrial bioenergetics.

Chelerythrine in vitro inhibited the proliferation of androgen-independent prostate cancer DU145 and PC-3 cell lines [20]. The obtained results indicated significant inhibition by chelerythrine in vitro invasion and metastasis of androgen-independent prostate cancer cells, at least in part, through the regulation of the protein expression of MMP/TIMP and disturbance of NF-κB activation. Cytotoxic activity of various alkaloids, e.g., sanguinarine, chelerythrine, and chelidonine was investigated against human osteosarcoma U2OS cancer cell line. Reference [21] investigated the antiproliferative effects of five alkaloids—sanquinarine, chelerythrine, chelidonine, sanguilutine, and chelilutinein on human malignant melanoma A-375 and SK-MEL-2 cell lines [22]. The investigated alkaloids have strong anti-proliferative effect and therefore have a high potential for the development of more efficient anti-melanoma therapies. The authors concluded that despite the similarity of investigated alkaloids molecular structures, the mechanism of cancer cell death induction is different for each alkaloid.

Anticancer activity of *Sanguinaria canadensis* extracts was in vitro investigated on human myelogenous leukemia K562 cell line [23].

In the present study, we investigated in vitro cytotoxic activities of isoquinoline alkaloids standards and plant extracts obtained from *Sanguinaria canadensis*, collected before, during, and after flowering, using human malignant melanoma cells (A375), human Caucasian malignant melanoma cell line (G361), and human malignant melanoma cell line (SKMEL3). These extracts were not previously tested against these cancer cell lines. Cytotoxic activity of plant extracts and alkaloid standards were compared with cytotoxicity obtained against these same cell lines for three anticancer drugs—etoposide, cisplatin, and hydroxyurea. To the best of our knowledge, the cytotoxic activity of *Sanguinaria canadensis* extracts against human melanoma cell lines were not tested before. This study provides a basis for future in-depth investigations on *Sanguinaria canadensis* extracts, which have the potential to be developed as an anticancer agent for the treatment of melanomas.

## 2. Results and Discussion

### 2.1. HPLC-DAD and LC-MS/MS Analysis of Alkaloid Standards and Plant Extracts

Alkaloid standards (see Table 1) were chromatographed in condition, based on the previously published method applied for determination of isoquinoline alkaloids, after appropriate modification [24]. Separation was performed on the Polar Reversed Phase (RP) column with the eluent system containing acetonitrile, water, and 0.04 ML^−1^ of 1-butyl-3-methylimidazolium tetrafluoroborate, in a gradient system described in the section “Experimental”. In this condition, symmetrical peaks and high theoretical plate numbers were obtained for all investigated alkaloids. Asymmetry factor (As) values obtained in the chromatographic system were: 1.01 for berberine, 1.07 for chelidonine, 1.11 for chelerythrine, 1.01 for protopine, and 0.99 for sanguinarine. Theoretical plate number per meter (N/m) values were —54,300 obtained for protopine and 490,200 for sanguinarine.

The equations of calibration curves obtained by means of the least square method for isoquinoline alkaloid standards, correlation coefficient (r), the limit of detection (LOD), and limit of quantification (LOQ) are given in Table S1. The MS parameters and conditions used for identification of the selected isoquinoline alkaloids in plant extracts are presented in Table S2.

In the same chromatographic system, alkaloids in extracts obtained from *Sanguinaria canadensis* collected before, during, and after flowering were analyzed. Typically, chromatograms obtained for plant extracts are presented in Figures S1 and S2. Chromatogram obtained for extracts from *Sanguinaria canadensis* collected during flowering was presented earlier [24]. The highest contents of sanguinarine and chelerythrine were found in all investigated extracts (Table 2). The content of sanguinarine in the extract from *Sanguinaria canadensis* collected before flowering was 4.8543 mg/g of dry plant material, while it increased significantly during the flowering of the plant and was 9.59 mg/g of dry plant material, and decreased after flowering to 6.92 mg/g of dry plant material. The content of chelerythrine also increased from 2.72 mg/g of dry plant material before flowering to 5.35 mg/g of dry plant material during flowering, while after flowering it was even higher and was 6.87 mg/g of dry plant material. The highest content of berberine was found during flowering (0.0125 mg/g of dry plant material), while the highest content of protopine was determined after flowering (0.1075 mg/g of dry plant material). The results showed that in order to obtain the highest content of alkaloids, *Sanguinaria canadensis* should be collected during flowering or after flowering.

The quantitative determination of selected isoquinoline alkaloids in plant extracts were performed in the same chromatographic conditions. The identities of the analyte peaks in plant extracts were confirmed by the comparison of their retention times and UV spectra with the retention times and MS and product ion MS/MS spectra of relevant alkaloid standards. This technique provides accurate mass measurement for both precursor and product ions, which gives a higher order mass identification. Isoquinoline alkaloids were quantified in extracts obtained from various parts of *Sanguinaria canadensis* collected before, during, and after flowering. Full MS and MS/MS spectra of between *m/z* 50–400 were acquired for each target compound. Moreover, because the ESI mass spectrum was obtained from an acidic solution that put the studied alkaloids into the positively charged quaternary nitrogen form, their peaks of highest mass corresponded to the actual molecular mass, [M + H]^+^. Hence, relevant isoquinoline alkaloids were identified based on the MS spectra for berberine (*m/z* = 335.7429), chelerythrine (*m/z* = 347.7489), chelidonine (*m/z* = 354.3920), protopine (*m/z* = 353.7655), and sanguinarine (*m/z* = 331.7065), respectively. Moreover, their presence in real plant samples regarding different morphological parts (before, after, or during flowering) was confirmed by MS/MS spectra, collision induced dissociation (CID) of the peak of the most intense ion. Examples of MS and MS/MS spectra obtained for the selected alkaloid from extracts are presented in Figure S3.

The identification of studied alkaloids in the plant extracts was based on the accurate mass measurements, the isotopic distribution of parent ions, and the study of their fragmentation patterns. In the MS/MS spectrum of berberine, the major product ion appeared at *m/z* = 319.7029 and corresponded to the elimination of methyl radical and CH_4_, respectively, from the methoxy substituent. The ion at *m/z* = 305.6823 was formed by continuous elimination of two methyl radicals and the ion at *m/z* = 304.1893 was formed by the loss of CH_3_OH from the precursor ion. The ions at *m/z* = 291.6987 and 277.6827 were then formed by the loss of CO from the ions at *m/z* = 319.7029 and *m/z* = 305.6823, respectively. Moreover, this sequential loss of a methyl radical and CO is the characteristic fragmentation pathway of this kind of alkaloid [25].

In the case of protopine, fragment ion at *m/z* = 205.7380 and 148.8711 in the MS/MS spectrum were generated by RDA (retro-Diels-Adler reaction) C ring opening [25], but given the presence of hydroxyl groups, the product ions at *m/z* = 336.1209 and *m/z* = 188.7681 were probably formed by loss of H_2_O from the molecular ion and from the *m/z* = 205.7380 ion.

In the case of sanguinarine, the MS/MS spectrum exhibited fragment ions at *m/z* = 316.6761 [M − CH_3_]^+^, *m/z* = 303.6993 [M − CO]^+^, and at *m/z* = 273.6853 [M − (CH_2_O + CO)]^+^. Based on the obtained results, similar fragmentation could be assumed for chelerythrine at *m/z* = 347.7489 as well as chelidonine at *m/z* = 354.3920, which were mainly fragmented to *m/z* = 303.6990 and *m/z* = 274.6920 [25].

### 2.2. Investigation of In Vitro Cytotoxic Activity of Isoquinoline Alkaloid Standards

The cytotoxic activity of alkaloid standards—sanguinarine, chelerythrine, chelidonine, berberine, and protopine were investigated using three melanoma cell lines (A375, G-361, SK-MEL-3) that differed in the original and mutated genes. Investigated alkaloids exhibited various cytotoxicity against tested human melanoma cell lines (Table 3). The very high cytotoxic activities were observed for sanguinarine and chelerythrine, with IC_50_ values below 0.55 µg/mL on all tested melanoma cells. Sanguinarine exhibited the highest cytotoxicity against A375 cell line (IC_50_ = 0.11 µg/mL), the lowest against SK-MEL-3 (IC_50_ = 0.54 µg/mL), while chelerythrine was most cytotoxic against SK-MEL-3 cells 3 (IC_50_ = 0.14 µg/mL) and showed the lowest cytotoxic activity against the G-361 melanoma cell line. Berberine showed moderate cytotoxic activity against all tested cell line. The lowest cell viability was observed after being treated by the berberine G-361 cells (IC_50_ = 21.02 µg/mL), highest viability was for A375 cells (IC_50_ = 51.60 µg/mL). For chelidonine and protopine, IC_50_ were more than 50 µg/mL. Due to the low solubility of these alkaloids in the cell culture medium, it was not possible to determine the viability of cells in the presence of their higher concentrations.

The cytotoxic properties of isoquinoline alkaloids were frequently confirmed against various types of cancer cells [3,19]. Our investigations indicated on very strong cytotoxic properties of sanguinarine and chelerythrine, against human melanoma cell lines.

### 2.3. Investigation of In Vitro Cytotoxic Activity of Sanguinaria canadensis Extracts

Cytotoxic activity of extracts obtained from *Sanguinaria canadensis* collected before, during, and after flowering was investigated against the same melanoma cell lines in the same conditions as the previously investigated alkaloid standards. All investigated plant extracts exhibited high cytotoxic activity against all tested cell lines (IC_50_ < 11 µg/mL). All *Sanguinaria canadensis* extracts were most active against the SK-MEL-3 cell line. Obtained IC_50_ values were very low (from 0.88 µg/mL after exposure to extract prepared from the plant before flowering to 1.36 µg/mL after exposure to extract prepared from the plant after flowering). Similar IC_50_ values were obtained after exposure of G-361 cells to all tested *Sanguinaria canadensis* extracts. IC_50_ values were from 1.22 µg/mL to 1.60 µg/mL. Additionally, in this case, the extract obtained from the plant before flowering was slightly more active and the extract obtained from the plant after flowering was the least active. The slowly lower cytotoxic activity was observed after exposure of A375 cells to all *Sanguinaria canadensis* extracts. Additionally, against the cell line, the most cytotoxic was the extract obtained from the plant collected before flowering (IC_50_ = 4.09 µg/mL) and the lowest cytotoxicity was observed after being treated by the extract from plant collected after flowering (IC_50_ = 10.96 µg/mL).

For comparison of *Sanguinaria canadensis* cytotoxicity against normal human fibroblast, IC_50_ values were calculated (Table 3). IC_50_ obtained for fibroblasts were higher than those obtained after exposure of cancer G-361 and SK-MEL-3 cells to the same plant extracts.

Our results demonstrated that *Sanguinaria canadensis* extracts are cytotoxic to various human melanoma cell lines, with IC_50_ values ranging from 0.88 to 10.96 µg/mL. Interestingly, the cytotoxicity was greater than that of other plant extracts containing various cytotoxic compounds, including alkaloids [26,27,28,29,30,31,32,33,34,35,36].

The extract obtained from *Sanguinaria canadensis* collected before flowering exhibited the strongest cytotoxic properties against all tested melanoma cells.

The selectivity index (SI) values were also calculated for the investigated plant extracts (Table 3). Selectivity is the main problem of the drugs in clinical use for the cancer treatment. If the compound or their mixture as plant extract had more selectivity potency towards cancer cells than normal cells, that compound could be evaluated as an anticancer drug candidate for further research. For all *Sanguinaria canadensis* extracts against A375 cells, the obtained SI values were below 1.0, which indicated that the concentration of extracts for achieving therapeutic effect were higher than the concentration causing toxic effects. Whereas high SI values (SI > 3.0) were obtained for all tested extracts from *Sanguinaria canadensis* against G-361 and especially against SK-MEL-3 (SI > 4.0) cells. As viability of various tested melanoma cell lines are differently sensitive after exposure to the extracts, it proved that any therapy should be preceded by genetic tests of the melanoma type.

The antiproliferative effect of *Sanguinaria canadensis* extracts and cisplatin were also investigated on SK-MEL-3 cells, using the BrdU assay, which indicated the rate of incorporation of 5′-bromo-2′-deoxy-uridine (BrdU) during DNA biosynthesis. Extracts obtained from *Sanguinaria canadensis* and cisplatin, dose-dependently inhibited BrdU incorporation in SK-MEL-3 cells. IC_50_ values obtained for *Sanguinaria canadensis* extracts and cisplatin are presented in Table 3. All investigated extracts exhibited antiproliferative activities significantly higher than that of the reference agent cisplatin on the SK-MEL-3 cells. A 50% inhibition was seen at 24.84 µg/mL of cisplatin but at 0.56 µg/mL for *Sanguinaria canadensis* collected before flowering to 0.93 µg/mL for *Sanguinaria canadensis* collected after flowering. These results indicated that *Sanguinaria canadensis* extracts exhibited high antiproliferative effect and confirmed their anticancer potential.

Among the quantified alkaloids, the extracts contained the highest contents of chelerythrine and sanguinarine. These alkaloids also showed the highest cytotoxicity, therefore, they certainly played a very important role in the cytotoxicity of *Sanguinaria canadensis* extracts. No significant correlation between extract alkaloid constituents and cell viability and proliferation was obtained, suggesting that unidentified constituents in the plant might additionally influence these cytotoxic activity. Similar results were described by Senchina et al. [37]. They investigated in vitro proliferation of chronic myelogenous leukemia K562 cells after exposure to *Sanguinaria canadensis* extracts obtained from various parts of the plant. Proliferation of K562 cells negatively correlated with both chelerythrine and sanguinarine contents.

### 2.4. Comparison of In Vitro Cytotoxic Activity of Alkaloid Standards, Sanguinaria canadensis Extracts with Cytotoxic Activity of Anticancer Drugs

For comparison of cytotoxic activity of alkaloid standards and plant extracts, IC_50_ values were also determined in the same conditions for three anticancer drugs—etoposide, cisplatin, and hydroxyurea (Table 3). Nearly almost all IC_50_ values obtained on all tested melanoma cell lines exposed on anticancer drugs were higher than the IC_50_ obtained after treating these same cells by *Sanguinaria canadensis* extracts and by sanguinarine and chelerythrine standards. The highest cytotoxic activity of anticancer drugs against melanoma cells was obtained for cisplatin. IC_50_ values obtained for the drug were 10.62 µg/mL obtained for the A375 cells to 14.42 µg/mL for the SK-MEL-3 cell line. However, these values in almost all cases were higher than the IC_50_ values obtained for the *Sanguinaria canadensis* extracts. Only IC_50_ values obtained for extracts from the plant collected after flowering and for cisplatin against A375 cells were similar. The IC_50_ for sanguinarine and chelerythrine standards were significantly lower than IC_50_ obtained for cisplatin, against all tested melanoma cell lines.

The comparison of the cytotoxic activity of *Sanguinaria canadensis* extracts and the alkaloids contained in them, with the cytotoxicity of anticancer drugs, indicates their high cytotoxicity and the possibility of their potential application in further investigations on the search for new drugs that could be used in the therapy of melanomas.

### 2.5. Comparison of In Vitro Cytotoxic Activity of Sanguinaria canadensis Extracts with Cytotoxic Activity of Various Plant Extracts

*Sanguinaria canadensis* extracts exhibited highly cytotoxic activity compared to previously published investigations of various plant extracts cytotoxicity against different melanoma cells [26,27,28,29,30,31,32,33,34,35,36]. IC_50_ values obtained after exposure of A375 cells to *Sanguinaria canadensis* extracts were from 4.09 to 10.96 µg/mL, values after exposure of G-361 cells to the plant extract were from 1.22 to 1.60 µg/mL, and after exposure of SK-MEL-3 to the same extracts were from 0.88 to 1.36 µg/mL. IC_50_ values obtained for previously investigated plant extracts against various melanoma cells were often significantly higher and were 2.44 µg/mL obtained after treating of mouse melanoma cell line B16-F0 by *Prosopis strombulifera* extract to 87.28 µg/mL obtained against human melanoma cell line A375, exposed to the *Moringa oleifera* extract. The comparison confirmed the very high cytotoxic activity of *Sanguinaria canadensis* extracts against the tested melanoma cells.

## 3. Experiment

### 3.1. Chemicals and Plant Materials

Acetonitrile (MeCN), methanol (MeOH), 1-butyl-3-methylimidazolium tetrafluoroborate of chromatographic quality were obtained from E. Merck (Darmstadt, Germany), dimethyl sulfoxide (DMSO) was obtained from Sigma-Aldrich (St. Louis, MO, USA).

Alkaloid standards (sanguinarine, chelerythrine, protopine, and chelidonine) were purchased from Chem Faces Biochemical Co. Ltd. (Wuhan, China). Berberine was purchased from Sigma-Aldrich (St. Louis, MO, USA).

Plant materials were collected before flowering in March, during flowering in April, and after flowering in June 2020. Plants organs were cut into pieces and dried at ambient temperature for 1–2 weeks.

### 3.2. Apparatus and HPLC-DAD Conditions

Analysis was performed using an LC-20AD Shimadzu (Shimadzu Corporation, Canby, OR, USA) liquid chromatograph. The chromatograph was equipped with a Shimadzu 364 SPD-M20A detector (Shimadzu Corporation, Canby, OR, USA). Detection was carried out at a wavelength of 240 nm. All chromatographic measurements were controlled by a CTO-10ASVP thermostat (Shimadzu Corporation, Canby, OR, USA). The eluent flow rate was 1.0 mL/min. Extracts were injected into the columns using the Rheodyne 20 µL injector. The DAD detector was set in the 200–800 nm range. Data acquisition and processing were carried out with a LabSolutions software (Shimadzu Corporation, Kyoto, Japan). Analysis was performed on Synergi Polar RP 80A column (150 × 4.6 mm, 5 µm). The mobile phase was composed out of 0.04 ML^−1^ 1-butyl-3-methylimidazolium tetrafluoroborate in water (solvent A) and 1-butyl-3-methylimidazolium tetrafluoroborate in acetonitrile (solvent B) in gradient elution: 0–20 min, 25% B; 20–30 min, 25–32% B; 30–40 min, 32–40% 373 B, and 40–60 min, 40% B. Flow rate was 1 mL/min. Calibration curves were constructed by analyzing the alkaloid standards at eight concentrations, ranging from 0.001 to 0.2 mg/mL. The calibration curves were obtained by means of the least square method. The limit of detection (LOD) and limit of quantification (LOQ) obtained for alkaloids were calculated according to the formulas: LOD = 3.3 (SD/S), and LOQ = 10 (SD/S), where SD is the standard deviation of response (peak area) and S is the slope of the calibration curve. HPLC analyses of alkaloid standards and plant extracts were repeated three times.

### 3.3. HPLC-MS/MS

Determination and identification of selected alkaloids was carried out using an HPLC Agilent 1290 Series system (Agilent Technologies, Waldbronn, Germany), equipped with an ESI interface, a 6540 UHD accurate mass Q-TOF detector, and Mass Hunter software for data collection and instrumental control. Chromatographic XDB-C18 column (4.6 mm × 50 mm, 1.8 µm, Agilent Technologies, Waldbronn, Germany) was maintained at 20 ± 0.5 °C. The injected sample volume was 20 µL, while the mobile phase was composed of ACN + 0.1% HCOOH (30:70, *v/v*) dosed at a flow rate of 0.6 mL/min. Quadrupole time-of-flight mass spectrometric analyses were performed using electrospray ion source operating in positive ion mode (ESI(+)), with the following set of operation parameters—capillary voltage (CV), 3.5 kV; octopole voltage (OV), 800 V; skimmer voltage (SV), 50 V; drying gas temperature (DGT), 295 °C; shielding gas temperature (SGT), 315 °C; mass spectra were recorded across the range mass range 40–370 *m/z*; and a fragmentor voltage (FV) of 195 V. Nitrogen was used as a drying (7 L/min) and nebulizng (40 psig) gas. The HPLC–MS data were acquired and quantified with the use of MassHunter Workstation software. The data were further processed using Microsoft Excel. The instrument was operated in extracted ion chromatogram (EIC) and product ion modes, respectively.

### 3.4. Extraction Procedure

The previously described procedure of alkaloids extraction from plant materials was applied after minor modifications [38,39]. Weighted samples (5 g) of each plant were macerated with 100 mL ethanol for 72 h and continuously extracted in an ultrasonic bath for 5 h. Extracts were filtered, the solvent was evaporated under vacuum, and the residues were dissolved in 30 mL of 2% sulfuric acid, and defatted with diethyl ether (3 × 40 mL). The aqueous layers were subsequently basified with 25% ammonia to a pH of 9.5–10, and the alkaloids were extracted with chloroform (3 × 50 mL). After evaporation of the organic solvent, the dried extracts were dissolved in 5 mL MeOH, prior to HPLC analysis.

### 3.5. Investigation of Cytotoxic Activity

#### 3.5.1. Investigation of Cell Viability

Cytotoxicity of the plant extracts, berberine, chelerythrine, chelidonine, protopine, and sanguinarine were examined against a panel of three melanoma cell lines (A375, G-361, SK-MEL-3) characterized by different degrees of genetic complexity. Human skin fibroblasts (CRL-1474) were used in order to evaluate the effect of *Saguinaria canadensis* extracts against normal (i.e., non-cancerous) cells. The investigated cell lines were obtained from American Type Culture Collection (ATCC; Manassas, VA, USA). A375 and CRL-1474 cells were cultured in Dulbecco’s Modified Eagle’s Medium (DMEM) (Sigma Aldrich, St. Louis, MO, USA), supplemented with 10% heat inactivated fetal bovine serum (FBS), penicillin (100 U/mL), and streptomycin (100 µg/mL). Human melanoma SK-MEL-3 and G-361 cells were maintained in McCoy’s 5A Medium (Sigma Aldrich, St. Louis, MO, USA) supplemented with 15% (for SK-MEL-3) or 10% (for G-361) FBS, penicillin (100 U/mL), and streptomycin (100 µg/mL). The cells were maintained at 37 °C in a 5% CO_2_ atmosphere. The dried plant extracts and standards (magnoflorine, palmatine, and berberine) were dissolved in DMSO, in order to obtain stock solutions at concentrations of 50 mg/mL. At the day of the experiment, the suspension of cells (1 × 105 cells/mL) in the respective medium was applied to a 96-wellplate at 100 µL per well. After 24 h of incubation, the medium was removed from the wells and replaced by increasing concentrations of plant extracts or standards in medium containing 2% FBS. The control cells were only cultured with a medium containing 2% FBS. Cytotoxicity of DMSO was also checked at concentrations present in respective dilutions of stock solutions. After 24 h of incubation, 15 µL MTT working solution (5 mg/mL in PBS) was added to each well. The plate was incubated for 3 h. Subsequently, 100 µL of 10% SDS solution was added to each well. Cells were incubated overnight at 37 °C to dissolve the precipitated formazan crystals. The concentration of the dissolved formazan was evaluated by measuring the absorbance at λ = 570 nm, using a microplate reader (Epoch, BioTek Instruments, Inc., Winooski, VT, USA). Two independent experiments were performed in triplicates. The results of the MTT assay were expressed as mean ± SD. DMSO in the concentrations present in the dilutions of stock solutions did not influence the viability of the tested cells. IC_50_ values of the investigated extracts and alkaloid standards were calculated using the IC_50_ calculator (www.IC50.tk (accessed on 22 January 2021)).

To determine the selectivity index (SI), IC_50_ values for human normal fibroblasts was obtained in the same conditions as that used for cancer cell lines. Selectivity index of each compound was determined by calculating the ratio of IC_50_ values obtained for human normal fibroblasts and cancer cell lines.

#### 3.5.2. Antiproliferative Effect of *Sanguinaria canadensis* Extracts against SK-MEL-3 Cells

The antiproliferative effect of *S. canadensis* extracts was measured by means of the BrdU assay, which indicates the rate of incorporation of 5′-bromo-2′-deoxy-uridine (BrdU) during DNA biosynthesis. In brief, SK-MEL-3 cells were plated in 96-well plates (NUNC, Roskilde, Denmark) at the density of 5 × 104 cells/mL. The next day, cells were exposed to increased concentrations (0.25–100 µg/mL) of *S. canadensis* extracts or cisplatin or a fresh culture medium. Cell proliferation was examined after 24 h incubation, according to the manufacturer’s protocol (Cell Proliferation ELISA BrdU, Roche Diagnostics GmbH, Penzberg, Germany). IC_50_ values of the investigated extracts and cisplatin were calculated using IC_50_ calculator [www.IC50.tk (accessed on 11 March 2021)].

## 4. Conclusions

Extracts obtained from the plant material collected before, during, and after flowering contained different isoquinoline alkaloids contents and showed various cytotoxic activity.

The highest activity against all tested melanoma cell lines was found for sanguinarine and chelerythrine. IC_50_ values obtained after exposure of all tested melanoma cells on sanguinarine and chelerythrine were below 0.55 µg/mL.

Extracts obtained from *Sanguinaria canadensis* exhibited very cytotoxic activity against all tested human melanoma cell lines (A375, G-361, SK-MEL-3). To the best of our knowledge, the cytotoxicity of the extracts are not yet investigated against cell lines tested by us.

The investigated plant extracts exhibited the highest cytotoxicity against SK-MEL-3. The highest cytotoxic activity against all tested cell lines was found for extracts prepared from *Sanguinaria canadensis* collected before flowering.

*Sanguinaria canadensis* extracts compared with extracts obtained from various plants tested as anticancer agents seems to be very promising for further investigations.

The extracts had a selective cytotoxicity with a high selectivity index (SI > 2) against G-361, SK-MEL-3 cell lines and could be considered to be potential new selective anticancer agents against selected melanoma cells.

In nearly all cases, sanguinarine, chelerythrine, and plant extracts showed higher cytotoxic activity against all tested melanoma cells, as compared to anticancer drugs—etoposide, cisplatin, and hydroxyurea. Such comparisons were very rarely made for plant extracts, but were not applied for *Sanguinaria canadensis* extract. It evidently indicated a very high cytotoxicity potential against the melanoma cells of the alkaloids and *Sanguinaria canadensis* extracts.

Based on these results, the investigated plant extracts obtained from *Sanguinaria canadensis* could be recommended for further in vivo experiments. Investigated extracts and alkaloids contained therein might be developed as a new candidate anticancer agent for treatment of human melanoma.

## Figures and Tables

**Table 1:** Physicochemical properties of the selected alkaloids.

Alkaloid	Chemical Structure	Molar Mass (g mol^−1^)	pKa	Log P
Berberine	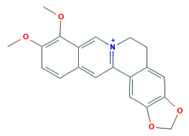	336.36	−4.4	−1.3
Chelerythrine	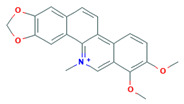	348.37	−4.4	−0.88
Chelidonine	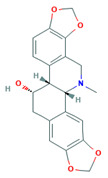	353.36	5.73	2.05
Protopine	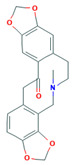	353.36	4.95	2.59
Sanguinarine	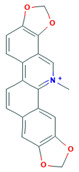	332.09	−4.5	−0.94

**Table 2:** Retention times of isoquinoline alkaloid standards and contents of isoquinoline alkaloids in *Sanguinaria canadensis* extracts [24].

Alkaloid	Contents of Alkaloids in Plant Extracts Obtained from *Sanguinaria canadensis* mg/g of Dry Plant Material		
	Before Flowering	During Flowering	After Flowering
Berberine	0.0058 (±0.0003)	**0.0125** (**±0.0010)**	0.0091 (±0.0007)
Chelerythrine	2.7224 (±0.0897)	5.3470 (±0.2018)	**6.8722** **(±0.1867)**
Chelidonine	-	<LOQ	-
Protopine	<LOQ	0.0141 (±0.0008)	**0.1075** **(±0.009)**
Sanguinarine	4.8543 (±0.1207)	**9.5899** (**±0.2302)**	6.9195 (±0.1624)

<LOQ—value below limit of quantification; - peak not identified. The highest content of a single alkaloid in extracts is marked in Bold.

**Table 3:** Selectivity index (SI), cytotoxic activity, expressed as IC_50_ values of the investigated extracts, alkaloid standards, and anticancer drugs against three melanoma cell lines (A375, G361, SK-MEL-3).

Plant Extracts and Alkaloid Standards	IC_50_ [µg/mL] ± SD for Cell Viability				Selectivity Index (SI) ** against A375/G-361/SK-MEL-3 Cell Lines	IC_50_ [µg/mL] ± SD for Cell Proliferation Measured by BrdU Incorporation in SK-MEL-3 Cells
	A375	G-361	SK-MEL-3	Fibroblasts	SK-MEL-3	
*Sanguinaria canadensis* before flowering	4.09 ± 0.25	1.22 ± 0.21	0.88 ± 0.06	3.78 ± 0.71	0.92/3.1/4.3	0.56 ± 0.02
*Sanguinaria canadensis* during flowering	5.08 ± 0.47	1.47 ± 0.07	1.13 ± 0.10	4.62 ± 0.92	0.91/3.1/4.1	0.68 ± 0.04
*Sanguinaria canadensis* after flowering	10.96 ± 0.50	1.60 ± 0.24	1.36 ± 0.18	6.27 ± 0.69	0.57/3.9/4.6	0.93 ± 0.22
Berberine	51.60 ± 4.84	21.02 ± 3.25	41.05 ± 5.36			
Chelerythrine	0.19 ± 0.04	0.46 ± 0.07	0.14 ± 0.004			
Sanguinarine	0.11 ± 0.003	0.17 ± 0.02	0.54 ± 0.16			
Chelidonine *	>50	>50	>50			
Protopine *	>50	>50	>50			
Etoposide	92.34 ± 4.58	52.32 ± 3.86	>200			
Cisplatin	10.62 ± 1.04	11.53 ± 1.46	14.42 ± 1.61			24.84 ± 3.60
Hydroxyurea	>200	>200	>200			

* Due to low solubility of chelidonine and protopine in the cell culture medium, it was not possible to determine the viability of cells in the presence of the higher concentrations of alkaloids. ** SI = IC_50_ of the respective extract against human normal fibroblasts/IC_50_ of the respective extract against the melanoma cell line (A375/G-361/SK-MEL-3).

## Data Availability

Data is contained within the article.

## Supplementary Materials

The following are available online, Figure S1: Chromatogram obtained from *Sanguinaria canadensis* extract collected before flowering, Figure S2: Chromatogram obtained from *Sanguinaria canadensis* extract collected after flowering, Figure S3: MS spectrum. Table S1: Equation of calibration curve, correlation coefficients (r), limit of detection (LOD) and limit of quantification (LOQ) values, Table S2: MS parameters and conditions used for determination and identification of selected alkaloids in plant samples.

## Sample Availability

Samples of the compounds are not available.
