# Supplementary material for: Determination of Cytotoxic Activity of Sanguinaria canadensis Extracts against Human Melanoma Cells and Comparison of Their Cytotoxicity with Cytotoxicity of Some Anticancer Drugs
Source: Molecules. 2021 Mar 20;26(6):1738. doi: 10.3390/molecules26061738 (PMC8003779; doi:10.3390/molecules26061738)
Supplement: Supplementary file 1 [file molecules-26-01738-s001.pdf]

Table S1. Equation of calibration curve, correlation coefficients (r), limit of detection (LOD) and limit of quantification (LOQ) values.

Alkaloid	**t_R (min)**	Equation of calibration curve	r	LOD [mg/mL]	LOQ [mg/mL]
Berberine	35.48	y = 70984852x − 3300769	0.9990	0.0128	0.0422
Chelerythrine	42.57	y = 57154058x + 12978	0.9989	0.0086	0.0260
Chelidonine	19.33	y = 16505788x – 35518	0.9991	0.0111	0.0336
Protopine	14.18	y = 525340599x + 98298	0.9983	0.0222	0.0673
Sanguinarine	36.82	y = 63958474x + 118255	0.9994	0.0060	0.0182

Table S2. MS parameters and conditions used for determination and identification of selected alkaloids in plant samples.

Compound	Elemental composition	t_R (min)	Polari ty	Theoretica l (*m/z*)	Measured (*m/z*)	Major fragment ions	Error (ppm)	ID Score [%]	Collision energy (eV)	Fragmentor
Berberine	$C_{20}H_{18}NO_4$ $[M+H]^+$	3.12	ESI+	335.7426	335.7429	319.7029<br>305.6823<br>304.1893<br>291.6987<br>277.6827	-0.41	99.18	20	195
Chelerythrine	$C_{21}H_{18}NO_4$ $[M+H]^+$	3.98	ESI+	347.7491	347.7489	331.7071<br>303.6990<br>274.6920<br>231.6968	1.19	99.56	20	195
Chelidonine	$C_{20}H_{20}NO_5$ $[M+H]^+$	1.94	ESI+	354.3922	354.3920	336.6503<br>303.6990<br>274.6920<br>189.4355	-1.17	99.90	20	195
Protopine	$C_{20}H_{20}NO_5$ $[M+H]^+$	1.68	ESI+	353.7653	353.7655	336.1209<br>274.6716<br>205.7380<br>188.7681<br>148.8711	-0.93	99.15	20	195
Sanguinarine	$C_{20}H_{14}NO_4$ $[M+H]^+$	2.75	ESI+	331.7068	331.7065	316.6761<br>303.6993<br>288.6723<br>273.6853<br>245.7012	1.26	99.34	20	195

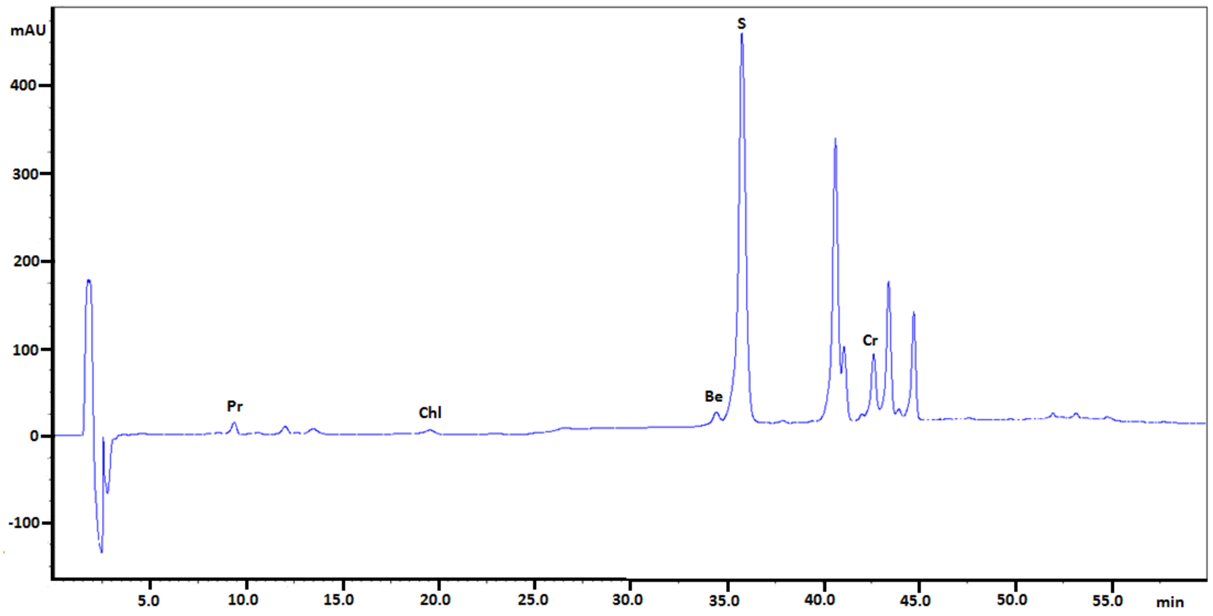

Figure S1. Chromatogram obtained from *Sanguinaria canadensis* extract collected before flowering. Be: Berberine; Cr: Chelerythrine; Chld: Chelidonine; Pr: Protopine; S: Sanguinarine.

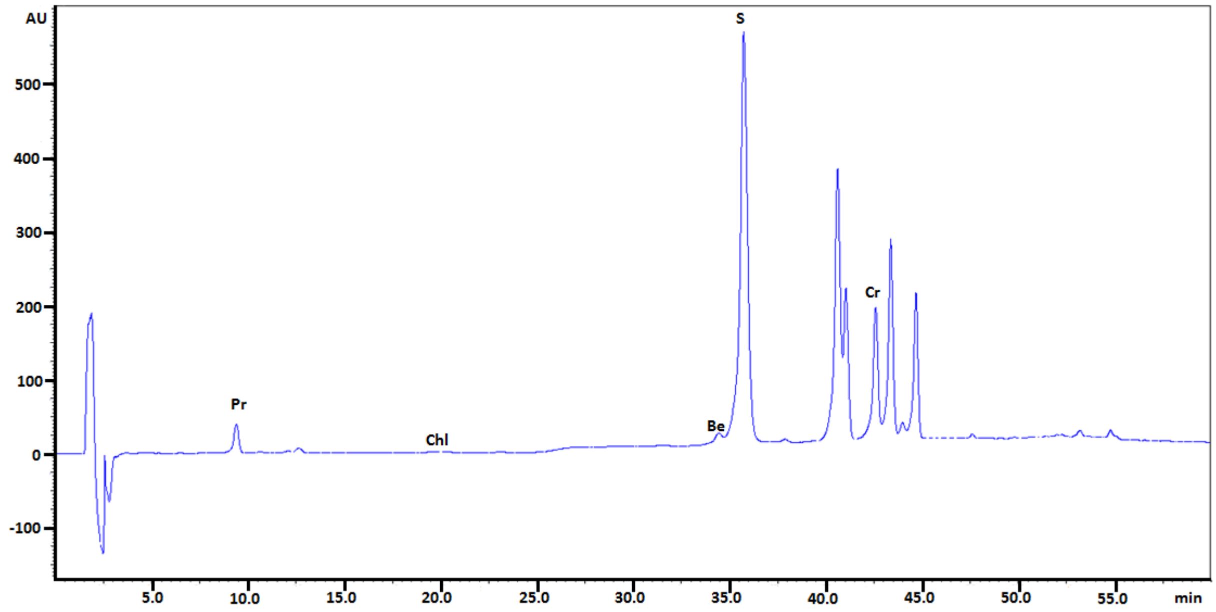

Figure S2. Chromatogram obtained from *Sanguinaria canadensis* extract collected after flowering. Be: Berberine; Cr: Chelerythrine; Chld: Chelidonine; Pr: Protopine; S: Sanguinarine

a)

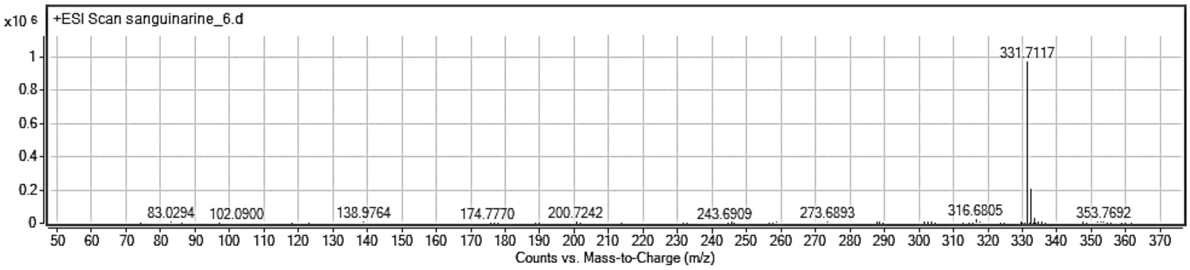

b)

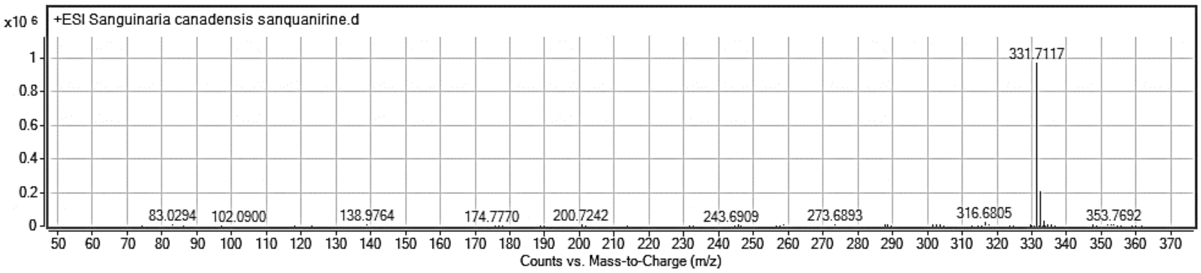

c)

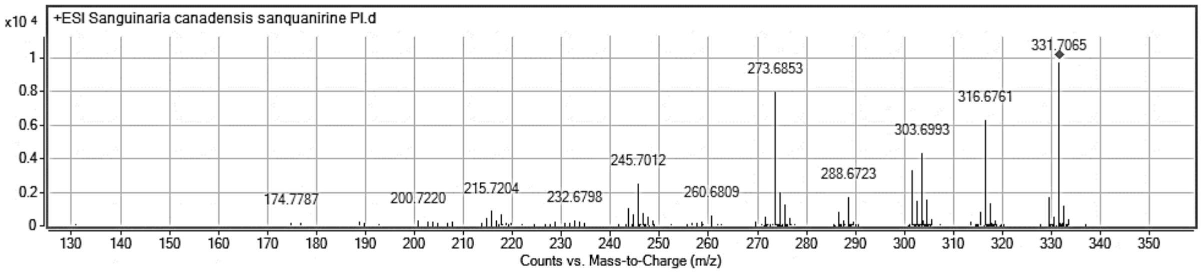

Figure S3. MS spectrum obtained for a) standard of sanguinarine, b) sanguinarine from *Sanguinaria canadensis* collected during flowering extract, c) MS/MS spectrum of sanguinarine from *Sanguinaria canadensis* collected during flowering extract. *Experimental conditions:* mobile phase composition: 0.1% HCOOH (A), ACN (B) 70:30 (*v/v*), flow rate: 0.6 mL/min, autosampler temperature 20ºC, drying gas flow rate 7.0 L/min; shielding gas flow rate 5.0 L/min; nebulizer gas pressure 40 psi; skimmer voltage 50 V, octopole voltage 800 V; capillary voltage 3500 V; drying gas temperature 295°C; shielding gas temperature 315°C; fragmentor voltage 195 V; CID energy in both MS and MS/MS were 5 and 20 eV.
